# Incidence and Risk Factors of Contrast-Induced Acute Kidney Injury in Sudanese Patients Undergoing Coronary Angiography: A Descriptive Prospective Study

**DOI:** 10.7759/cureus.21876

**Published:** 2022-02-03

**Authors:** Mohira A Abdalla, Kannan O Ahmed, Bashir A Yousef

**Affiliations:** 1 Department of Clinical Pharmacy, Faculty of Pharmacy, University of Khartoum, Khartoum, SDN; 2 Department of Clinical Pharmacy, Faculty of Pharmacy, University of Gezira, Wad Madani, SDN; 3 Department of Pharmacology, University of Khartoum, Khartoum, SDN

**Keywords:** sudan, coronary artery disease, contrast-induced acute kidney injury, contrast medium, coronary angiography

## Abstract

Background: Contrast-induced acute kidney injury (CI-AKI) has been reported to be the third most common cause of hospital-acquired acute kidney injury (AKI) and is associated with significant morbidity and mortality.

Objectives: This study aimed to assess the incidence and risk factors of CI-AKI in Sudanese patients undergoing coronary angiography.

Methods: The study was a descriptive, prospective, hospital-based study conducted at Alshaab Hospital between January and April 2019. A total of 163 patients undergoing coronary angiography were enrolled, and a checklist was used to collect data. Chi-square and regression analyses were performed to identify the risk factors of CI‑AKI. CI‑AKI was defined as a rise in serum creatinine ≥25% above baseline within three days after percutaneous coronary angiography (PCA).

Results: CI‑AKI incidence in patients undergoing coronary angiography was 31.9% (52/163). Among independent variables, diabetes mellitus, the type of coronary angiography, and the volume of contrast medium were statistically significantly (p-value ˂ 0.05) associated with CI-AKI development. Logistic analysis showed that diabetes (odds ratio (OR): 15.2; 95% CI: 6.771-33.974; p = 0.001), percutaneous coronary intervention (PCI) (OR: 2.4; 95% CI: 1.168-4.901; p = 0.017), and the volume of contrast medium (OR 2.1; 95% CI: 1.440-3.054; p = 0.001) were independent predictors correlated to CI‑AKI development.

Conclusion: The incidence of CI-AKI is 31.9%, and diabetes mellitus, PCI, and high volume of contrast medium are associated with a higher risk of CI-AKI in patients undergoing coronary angiography.

## Introduction

Coronary artery disease (CAD) is a disorder in which atheroma, thrombosis, or spasm of coronary arteries obstructs the vascular supply to the heart. This may reduce the supply of oxygenated blood to heart tissue to the point that myocardial ischemia occurs, which, if severe or prolonged, can result in cardiac muscle cell death [1]. CAD is one of the leading causes of death worldwide and remains a substantial contributor to morbidity, mortality, and healthcare expenditure [2]. CAD is currently classified to either chronic coronary syndrome (CCS) as stable angina or acute coronary syndrome (ACS), which is further classified to non-ST segment elevation ACS (NSTACS) or ST-segment elevation ACS (STACS) [3]. Management of CAD generally depends on different factors, such as whether it is ACS or CCS, the degree of coronary occlusion, and the onset of symptoms, and it includes but is not limited to immediate reperfusion through thrombolytic therapy or percutaneous coronary intervention (PCI), antiplatelets, and anticoagulants, and certain cases of CAD can be approached by coronary artery bypass grafting surgery [4].

Coronary angiography is the procedure that uses an intravascular injection of contrast medium to examine blood flow through the coronary arteries [5]. There are many classifications to contrast media depending on osmolality (low osmolar, iso-osmolar, and high osmolar contrast media), electronic charge (ionic and non-ionic contrast media), or iodination (iodinated and non-iodinated contrast media) [5]. Contrast medium administration can lead to severe acute kidney injury (AKI), known as contrast-induced AKI (CI-AKI). CI-AKI is the third most common cause of iatrogenic AKI [6] and has been associated with an increased risk of serious adverse events, such as renal failure, myocardial infarction, stroke, and death. Even minor increases in serum creatinine have been linked to more extended hospital stays and higher expenses [7].

According to reports, the incidences of CI-AKI ranged from 3% to 50%, depending on the demographic and risk factors. The risk of CI-AKI is less in individuals with normal renal function, but it increases in those with one or more risk factors [8]. So far, the incidence and risk factors of CI-AKI in Sudanese patients have not been investigated. Thus, this study aimed to assess CI-AKI in a Sudanese population undergoing coronary angiography.

## Materials and methods

Study design and setting

This study was a descriptive prospective hospital-based study. It was carried out in Alshaab Teaching Hospital, Khartoum, Sudan. The study population was patients undergoing percutaneous coronary angiography (PCA) at Alshaab Teaching Hospital from January to April 2019.

Participants and study size

The study participants are those who underwent PCA at Alshaab Teaching Hospital during the study period. A total coverage sampling was applied based on the inclusion and exclusion criteria. All admitted patients undergoing a single coronary angiography were included, whereas patients undergoing multiple coronary angiographies were excluded from this study.

Data collection methods

A pre-designed checklist was used as a data collection form, and it consisted of patients' demographics, including age and gender. The second part was clinical characteristics, including the type of comorbid diseases and concurrent drugs used by the patient. The third part included the type of coronary angiography (diagnostic or percutaneous coronary intervention (PCI)) and the type and injected volume of the contrast medium. The fourth part was about serum creatinine (SCr) measurements on admission as a baseline and daily after coronary angiography for three days. CI‑AKI was operationally defined as an increase in SCr ≥25% or ≥44.2 μmol/L (0.5 mg/dl) above baseline in three days after exposure to contrast medium [5].

Statistical analysis

Data were analyzed by the IBM Statistical Package for the Social Sciences (SPSS), version 22.0 (IBM Corp., Armonk, NY). The number and percentages of study variables were represented in frequency tables. Chi-square test was used to test the statistical differences, and logistic regression analysis was conducted to identify the risk factors for developing CI-AKI. P-value ≤ 0.05 was considered to indicate the statistical significance.

## Results

The total number of the included patients was 163. As shown in Table 1, most patients were males (n = 109, 66.9%) and the dominant population (n = 135, 82.8%) was aged 45-65 years, with a mean age of 60.67 ± 10.72 years. Clinically, ST-segment elevation myocardial infarction (STEMI) was represented in 52.2% of patients, whereas non-ST-segment myocardial infarction (NSTEMI) was found in 42.3% (Table 1). The majority of patients (66.9%) had normal baseline SCr (≤13 mg/dl). Regarding comorbid diseases, hypertension and diabetes were found in 47.2% and 36.8% of patients, respectively. The highest frequency regarding concurrent medications was 95.7% for non-steroidal anti-inflammatory drugs (NSAIDs), and 66.3% and 16.6% for angiotensin-converting enzyme inhibitors (ACEIs) and angiotensin receptor blockers (ARBs) (Table 1).

**Table 1:** Distribution of socio-demographic and clinical characteristics, and the usage of the nephrotoxic medications among the study sample (n = 163). SD, standard deviation; CAD, coronary artery disease; STEMI, ST-segment elevation myocardial infarction; NSTEMI, non-ST-segment myocardial infarction; SCr, serum creatinine; NSAIDs, non-steroidal anti-inflammatory drugs; ACEIs, angiotensin-converting enzyme inhibitors; ARBs, angiotensin receptor blockers; CI-AKI, contrast-induced acute kidney injury.

Demographic and clinical data	Number (frequency %)
Gender	
Male	109 (66.9)
Female	54 (33.1)
Age (years)	
˂45	3 (1.9)
45-65	135 (82.8)
66-75	16 (9.8)
>75	9 (5.5)
Age, mean ± SD (years)	60.7 ± 10.7
Patient presentation (CAD)	
STEMI	85 (52.2)
NSTEMI	69 (42.3)
Unstable angina	9 (5.5)
Comorbid diseases	
Diabetes mellitus	60 (36.8)
Hypertension	77 (47.2)
Baseline serum creatinine (mg/dl)	
0-1.3	109 (66.9)
1.4-1.9	44 (27)
≥2	10 (6.1)
SCr, mean ± SD (mg/dl)	1.24 ± 0.32
Use of nephrotoxic drugs	
NSAIDs	156 (95.7)
ACEIs	108 (66.3)
ARBs	27 (16.6)
Diuretics	24 (14.7)
Development of CI-AKI	
Yes	52 (31.9)
No	111 (68.1)

With regard to the purpose of coronary angiography, it was indicated for treatment as PCI in 59.5% of patients, while it was prescribed in 40.5% for a diagnostic purpose (Table 2). Iohexol was used as the contrast medium for angiography in all patients (100%). However, the volume of injected iohexol varied, as 38.8% of patients were injected with 100-199 ml, whereas <100 ml, 200-299 ml, and ≥300 ml were used in 21.5%, 25.5%, and 13.3% of patients, respectively (Table 2).

**Table 2:** Distribution of the study sample according to the type of coronary angiography and the type and volume of injected contrast media (n = 163).

Variable	Number (frequency %)
Type of coronary angiography	
Diagnostic	66 (40.5)
Percutaneous coronary intervention	97 (59.5)
Type of contrast media	
Iohexol	163 (100)
Volume of injected contrast media (ml)	
<100	35 (21.5)
100-199	64 (38.8)
200-299	42 (25.5)
≥300	22 (13.3)

In the current study, CI‑AKI incidence in patients who underwent coronary angiography was 31.9% (52/163). Statistically, as shown in Table 3, there were no significant associations between CI-AKI and socio-demographics (gender and age), hypertension, and clinical presentation of patients. In contrast, diabetes mellitus was significantly associated with CI-AKI (p = 0.001). Usage of concurrent drugs was insignificantly associated with CI-AKI. Furthermore, the type of coronary angiography procedure is significantly associated with CI-AKI (p = 0.012), as more cases of CI-AKI were presented in patients with PCI. Besides, increased volume of injected iohexol was significantly associated (p = 0.001) with CI-AKI development (Table 3).

**Table 3:** Association between CI-AKI development and socio-demographics, clinical characteristics, and the usage of the nephrotoxic medications among the study sample (n = 163). CI-AKI, contrast-induced acute kidney injury; CAD, coronary artery disease; STEMI, ST-segment elevation myocardial infarction; NSTEMI, non–ST-segment myocardial infarction; NSAIDs, non-steroidal anti-inflammatory drugs; ACEIs, angiotensin-converting enzyme inhibitors; ARBs, angiotensin receptor blockers; PCI, percutaneous coronary intervention.

Variables	CI-AKI development		P-value
	Yes (number (%))	No (number (%))	
Gender			
Male	33 (20.2)	76 (46.6)	0.527
Female	19 (11.7)	35 (21.5)	
Age (years)			
˂40	2 (1.2)	1 (0.6)	
40-65	40 (24.6)	95 (58.3)	0.23
66-75	5 (3.1)	11 (6.7)	
>75	5 (3.1)	4 (2.4)	
Diabetes mellitus			
Diabetic	40 (24.5)	20 (12.3)	0.001
Non-diabetic	12 (7.4)	91 (55.8)	
Hypertension			
Hypertensive	29 (17.8)	48 (29.4)	0.135
Non-hypertensive	23 (14.1)	63 (38.7)	
Patient presentation (CAD)			
STEMI	31 (19)	54 (33.1)	
NSTEMI	17 (10.4)	52 (31.9)	0.208
Unstable angina	4 (2.5)	5 (3.1)	
Baseline serum creatinine (mg/dl)			
0-1.3	33 (20.2)	76 (46.6)	
1.4-1.9	13 (8)	31 (19)	0.144
≥2	6 (3.7)	4 (2.5)	
NSAIDs			
Used	49 (30.1)	107 (65.6)	0.525
Not used	3 (1.8)	4 (2.5)	
ACEIs			
Used	33 (20.2)	75 (46)	0.605
Not used	19 (11.7)	36 (22.1)	
ARBs			
Used	11 (6.7)	16 (9.8)	0.281
Not used	41 (25.2)	95 (58.3)	
Diuretics			
Used	9 (5.5)	15 (9.2)	0.524
Not used	43 (26.4)	96 (58.9)	
Type of coronary angiography			
PCI	38 (23.3)	59 (36.2)	0.016
Diagnostic	14 (8.6)	52 (31.9)	
Volume of contrast media (ml)			
<100	4 (2.5)	32 (19.6)	
100-199	19 (11.7)	44 (27)	0.001
200-299	15 (9.2)	27 (16.5)	
≥300	14 (8.6)	8 (4.9)	

When logistic regression analyses were performed to identify the predictors for CI-AKI risk factors, as demonstrated in Table 4, we found that diabetic patients were more likely to develop CI-AKI than nondiabetic patients by approximately 15 times (CI: 6.771-33.974; p = 0.001). Also, using coronary angiography procedure for PCI was more likely to cause CI-AKI than for diagnostic purposes by 2.4 times (CI: 1.168-4.901; p = 0.017). Furthermore, patients who received a larger volume of contrast media were more likely to develop CI-AKI two times than those who received a lower volume of the contrast medium (CI: 1.440-3.054; p = 0.001) (Table 4).

**Table 4:** Predicting the patient's risk factors for developing contrast-induced acute kidney injury by using binary logistic regression test (n = 163). B, logistic correlation coefficient; P, p-value; OR, odds ratio; CI, confidence interval; CAD, coronary artery disease; NSAIDs, non-steroidal anti-inflammatory drugs; ACEIs, angiotensin-converting enzyme inhibitors; ARBs, angiotensin receptor blockers.

Variables	B	P	OR	95% CI for OR	
				Lower	Upper
Gender	−0.233	0.527	0.527	0.400	1.598
Age	−0.279	0.344	0.757	0.425	1.348
Diabetes	2.719	0.000	15.167	6.771	33.974
Hypertension	0.504	0.137	1.655	0.853	3.214
Patient presentation (CAD)	0.221	0.441	1.247	0.712	2.184
Baseline serum creatinine	0.346	0.203	0.708	0.415	1.205
Use of NSAIDs	−0.493	0.783	0.529	0.611	2.833
Use of ACEIs	−0.182	0.352	0.606	0.834	1.663
Use of ARBs	0.466	0.434	0.283	1.593	3.729
Use of diuretics	0.292	0.460	0.525	1.340	3.299
Type of coronary angiography	0.872	0.017	2.392	1.168	4.901
Volume of contrast media	0.741	0.000	2.097	1.440	3.054

## Discussion

CI-AKI has attracted more attention after the wide application of angiography technology for managing CAD patients, as it is a complication associated with increased morbidity and mortality [5]. In the current study, CI‑AKI incidence was 31.9% in Sudanese patients who underwent coronary angiography, which is higher than previous studies conducted in China (7.2%), Austria (10.2%), and Canada (12.7%) [9-11].

The incidence of cardiovascular disease is higher in men than in women of similar age [12], as in this study, 66.9% of patients were males. On the other hand, women with CAD have been reported to have poor prognoses and higher cardiovascular risk factor profiles [13]. However, our findings indicated that gender and age were not significantly associated with the development of AKI, which is countered to previous studies that showed a significantly higher incidence of CI-AKI in females [14], and in elderly patients (age ≥75 years) [15]. This conflict may be due to the small sample size compared with other studies or may be related to genetic variation.

The exact mechanism behind CI‑AKI has not been fully understood. Several mechanisms may be involved in the pathogenesis of renal damage, including vasoconstriction of renal arteries, renal ischemia, tubular obstruction, inflammation, and oxidative stress induction [16]. There are many clinical characteristics such as diabetes mellitus, hypertension, and STEMI presentation that have been related to CI-AKI development [17,18]. In diabetic nephropathy patients, AKI episodes are linked to a higher likelihood of developing severe chronic kidney diseases [19], and those patients were more vulnerable to tubular injury caused by iohexol [20]. In our study, a significant association between the development of CI-AKI and diabetes mellitus has appeared, and this is consistent with other previous studies [2,21]. However, hypertension and CAD presentation were not associated with the development of CI-AKI, and this is countered to previous studies in which STEMI presentation was most strongly associated with the development of CI-AKI [22].

Concurrent medications may correlate to CI‑AKI development by contributing to renal tubule damage and disturbances of renal blood flow induced by contrast media [23]. Drugs such as ACEIs, ARBs, diuretics, NSAIDs, and metformin have been reported to increase CI-AKI risk for the patient undergoing coronary angiography, and withholding of these medications prior to administration of contrast medium may prevent CI-AKI development [24]. In the current study, concurrent use of NSAID, renin-angiotensin system blockers, and diuretics was not statistically correlated to CI-AKI. In contrast to previous studies that proposed ACEIs contributed to CI-AKI, particularly in patients with pre-existing renal impairment [2,25], another study showed that diuretics increase the risk of CI‑AKI through decreasing renal blood flow and increasing contrast medium toxicity [26].

Coronary angiography can be used for diagnostic purposes or therapeutic purposes as PCI [27]. In this study, the indication of coronary angiography procedure for PCI was more associated with the development of CI-AKI, which is consistent with other studies in which PCI risk for CI-AKI [15,28], and this may be due to the differences in procedure-related characteristics such as the catheter used, stent, and intra-aortic balloon pump [29]. Moreover, due to limited prophylactic strategies, CI‑AKI was mostly associated in patients with the emergency PCI procedure than the selective PCI procedure [14].

Iohexol is the only contrast medium available in this hospital; therefore, all patients in this study received it. Iohexol is a non-ionic, tri-iodinated contrast medium with a large volume of distribution, and more than 90% of iohexol is excreted unchanged by glomerular filtration within the first 24 hours. Therefore, iohexol should be used with extreme care in patients with severe functional disturbances of kidneys [20]. Also, dehydration is dangerous and may contribute to acute renal failure in patients with advanced vascular disease, diabetic patients, and susceptible nondiabetic patients. As a result, patients should be well-hydrated before and after receiving any contrast medium, including iohexol [20].

The current study showed that the volume of injected iohexol was different. In the hospital practice, we observed that determining the volume of contrast medium depends on doctors' experiences who do the angiography procedure, which may contribute to developing CI-AKI in some patients. Statistically, the volume of injected contrast is significantly associated with the development of CI-AKI; this is consistent with a study in which high doses of contrast media were considered a risk for CI-AKI [15] and associated with the severity of kidney injury and mortality [30]. In contrast, another study showed no statistical significance between the volume of injected contrast medium and CI-AKI development [14].

The current study has some limitations. Firstly, this was a single-institution study, so the result cannot be generalized to other hospitals. Secondly, we did not calculate the estimated glomerular filtration rate (eGFR) for patients due to the unavailability of body weight data. Thirdly, we did not study the treatment and preventive measures against CI-AKI and the clinical consequences after discharge from the hospital. Despite these limitations, this is the first study to assess the incidence and risk factors for CI-AKI in Sudanese patients, and we handled some challenges to reporting CI-AKI, which typically occurred after three days, and normally angiography does not require patients to be admitted, so some of the outpatients' data were missed. Thus, we recommend running multicenter studies with a larger population, including outpatients and inpatients, to confirm the exact incidence and risk factors of CI-AKI in Sudan.

## Conclusions

In conclusion, the study showed that CI‑AKI incidence in patients who underwent coronary angiography was high (31.9%). Diabetes mellitus, PCI, and high volume of contrast medium were significantly associated (p ˂ 0.05) with CI-AKI. They are considered the main risk factors for CI‑AKI devolvement in patients undergoing coronary angiography. Thus, more effective renal protection strategies during coronary angiography are needed, particularly in high-risk patients.
